# Influence of Dietary Advice Including Green Vegetables, Beef, and Whole Dairy Products on Recurrent Upper Respiratory Tract Infections in Children: A Randomized Controlled Trial

**DOI:** 10.3390/nu12010272

**Published:** 2020-01-20

**Authors:** Ellen van der Gaag, Ruben Brandsema, Rosan Nobbenhuis, Job van der Palen, Thalia Hummel

**Affiliations:** 1 Department of Pediatrics, Hospital Group Twente, 17609 PP Almelo, The Netherlands; r.nobbenhuis@zgt.nl; 2 Department of BMS, University Twente, 57522 NB Enschede, The Netherlands; ruben.brandsema@gmail.com (R.B.); j.vanderPalen@mst.nl (J.v.d.P.); 3 Medical Spectrum Twente, 17512 KZ Enschede, The Netherlands; t.hummel@mst.nl

**Keywords:** randomized controlled trial, children, dietary advice, vegetables, beef, whole dairy products, upper respiratory tract infections

## Abstract

Background: Since no treatment exists for children suffering from upper respiratory tract infections (URTIs) without immunological disorders, we searched for a possible tool to improve the health of these children. Aim: We evaluated whether dietary advice (based on food matrix and food synergy), including standard supportive care, can decrease the number and duration of URTIs in children with recurrent URTIs. Design and Setting: This study was a multicenter randomized controlled trial in two pediatric outpatient clinics in the Netherlands, with 118 children aged one to four years with recurrent URTIs. The dietary advice group received dietary advice plus standard supportive care, while the control group received standard supportive care alone for six months. The dietary advice consisted of green vegetables five times per week, beef three times per week, 300 mL whole milk per day, and whole dairy butter on bread every day. Portion sizes were age-appropriate. Results and Conclusion: Children in the dietary advice group had 4.8 (1.6–9.5) days per month with symptoms of an URTI in the last three months of the study, compared to 7.7 (4.0–12.3) in the control group (*p* = 0.028). The total number of URTIs during the six-month study period was 5.7 (±0.55) versus 6.8 (±0.49), respectively (*p* = 0.068). The use of antibiotics was significantly reduced in the dietary advice group, as well as visits to a general practitioner, thereby possibly reducing healthcare costs. The results show a reduced number of days with symptoms of a URTI following dietary advice. The number of infections was not significantly reduced.

## 1. Introduction

Upper respiratory tract infections (URTIs) are the most frequently diagnosed diseases in children, especially those aged one to six years. On average, the youngest children have six to eight infections per year [1,2,3]. Incidence varies seasonally with peaks in autumn and winter [1]. The severity and frequency of URTIs is inversely proportional to age, suggesting a build-up in immunity through repeated contact with causative viruses [2,4]. In children with frequent, long-lasting, or clinically severe infections, the clinician should consider an immunodeficiency [5]. Despite the majority of episodes being self-limiting, URTIs cause frequent and unnecessary prescription of antibiotics and absences from work for parents [6,7]. This antibiotic prescription incurs substantial costs for health care systems, especially when a substantial proportion consists of non-indicated and ineffective treatments [8]. The use of more than two antibiotic courses for a respiratory infection in the preceding year for a child is a good prognostic marker for response failure to the present treatment of the respiratory infection [9], implying a need to minimize antibiotic prescriptions for these respiratory infections in young children.

Multiple clinical and non-clinical trials investigated the role of varied single nutrient supplementation on (respiratory) infection. The results of these trials are unclear. For example, administration of vitamin D and iron showed conflicting results, whereas vitamin C administration showed a 18% decrease in duration of colds in children [10,11,12,13]. The lack of success of some supplementations may be due to a single nutrient model lacking synergy with other nutrients as part of a dietary lifestyle [14]. However, the effects of dietary interventions (with multiple components) on upper respiratory tract infections have not been widely investigated. Calatayud et al. studied the effect of a change toward the Mediterranean lifestyle in a prospective before–after comparison study. They found that the Mediterranean diet (fresh, locally produced, and non-processed food) had a significantly beneficial effect in patients with recurrent common cold and respiratory infections [15]. In a Nordic country (Sweden), with other locally produced food, a prospective population-based study showed no effect of this locally produced diet (Nordic diet) on URTIs. Only high physical activity was associated with lower URTI risk [16].

The Netherlands are located in between the Nordic and Mediterranean countries, which are the origins of the Dutch diet [17]. In the Netherlands, a large proportion of the total diet traditionally consists of dairy products [18], mostly cheese, milk, yoghurt, and butter. In 2009, we investigated the dietary patterns of children with recurrent URTI compared to control children. Questionnaires about dietary habits showed that children with URTIs consumed significantly fewer vegetables and beef compared to control children [19]. These products, however, have a high nutrient density [20]. Combining these findings, we selected four nutrient-dense food products to compose dietary advice fitting the latitude-specific food habits: Green vegetables, beef, whole milk, and butter. The diet was constructed using data from the Dutch Food Composition Database (NEVO) and the Netherlands Nutrition Centre (for age-appropriate amounts) for optimal nutritional values of iron, zinc, and vitamins A, C, D, and E (Table S1) [20,21].

Later, we performed a prospective before–after pilot study in children with dietary advice consisting of green vegetables, beef, and whole dairy products that were fresh and locally produced. We observed a drop in days with symptoms of the common cold of four days per month during the treatment period [22].

In the present study, we hypothesized that dietary advice could decrease the number and duration of upper respiratory tract infections compared with standard supportive care alone in children aged one to four years with recurrent upper respiratory tract infections. We also assessed whether the advice would reduce the number of days of parental absence from work and the number of prescribed antibiotics.

## 2. Materials and Methods

### 2.1. Study Design

We conducted a randomized controlled trial out in outpatient clinics of the Hospital Group Twente and the Medical Spectrum Twente. Both regional hospitals lie in the eastern part of the Netherlands. After inclusion, subjects were randomly allocated through minimization on a central remote computer, stratifying for age, day care attendance, and parental smoking. The QMinim Online Minimisation^®^ Program 0.3 [23] was used for this purpose. All pediatricians who included patients had to log into the central computer with a code, which was conducted in the presence of the child and their parents. The parents and the child witnessed, but could not influence, the process when the central computer allocated the group.

Both groups (intervention and control group) received standard supportive care for (recurrent) URTIs, e.g., painkillers, resting in bed, and antibiotics if necessary prescribed by their own, independent, general physician. The dietary advice group additionally received dietary advice. A detailed explanation (orally and a printed version) of the diet was offered only to the dietary advice group after randomization. Explanation about the dietary intervention took about 3–5 min. No behavioral approaches were offered or used during the study period. The control group did not receive dietary advice and were supposed to continue their usual dietary habits. The governing medical ethics committee of both hospitals approved the protocol (METC Enschede P14-39, NL50373.044.14). The trial was registered in the Dutch Trial Register (NTR4898, www.trialregister.nl).

### 2.2. Participants

Children 1–4 years, referred by their general physician with recurrent URTIs, were recruited if they had a minimum of 3 URTIs in the last 3 months. This had to be confirmed by the physician in the outpatient clinic by completing a daily questionnaire for a minimum of 1 month and asking the parents if this was representative for the last months. If more than two URTI episodes were noted and representative, they were included. If one or no URTI episode was noted, they were asked whether they had 3 episodes within 3 months. An URTI was defined as any combination of at least two of the following symptoms: Cough, fever, blocked or running nose, sore throat, wheeze, and/or ear discharge. A new URTI was counted after a minimum of two days with only one or zero symptoms; this definition is similar to that used in other studies [24].

Exclusion criteria included immunological deficiencies, cow’s milk allergy (use of an (extensively) hydrolyzed protein formula), intestinal malabsorption, prophylactic use of antibiotics for longer periods, disorders requiring a special diet, or any relevant congenital abnormality, anatomical abnormality, chromosomal disorder, or severe disease (altering metabolism and immune function). Written informed consent was obtained from parents and/or legal custodians.

### 2.3. Intervention

Participants in the dietary advice group were advised to follow dietary advice consisting of five times per week green vegetables at dinner, three times per week of beef at dinner, 300 mL whole cow’s milk daily, and whole dairy butter on each slice of bread. The advice was provided with age-specific portion sizes adhering to the guidelines and advice of the Netherlands Nutrition Centre (Table S2).

The pediatrician instructed parents during the visit in the outpatient clinic. The control group was not informed about the contents of the dietary advice. They continued their normal dietary habits consisting of the national advice by the Netherlands Nutrition Centre. The national advice only specifies not to eat processed or fatty meat; it provides no differentiation between types of vegetables and it suggests dairy intake should mainly come from regular or skimmed products [21]. A run-in period of one month was used before incorporation of the advice into daily life, which served as a baseline measurement of respiratory complaints. We scheduled contacts with the clinic at three and six months. To minimize seasonal influences, participants were included in 2 consecutive years all year round.

### 2.4. Data Collection

Baseline data and patient characteristics were collected around the inclusion period by the clinician. Parents recorded cough, fever, blocked or runny nose, sore throat, wheeze, respiratory distress, and/or ear complaints daily via a monthly diary for a run-in period of 1 month and for 6 months during the dietary intervention. Parents used self-diagnosis diaries to report the URTI symptoms. This is assumed to be the most reasonable approach to record common cold symptoms for clinical research studies [25,26].

Parents also reported contacts with physicians, antibiotics administered, and parental absence from work due to the illness of the child. Parents of children in the dietary advice group recorded their adherence to the dietary advice weekly (e.g., how many days a week did they drink whole milk, eat butter on bread, green vegetables, or beef). Adherence to the diet was expressed in percentages. These percentages were derived by dividing the number of portions patients provided, according to their diaries, by the total number of portions they were instructed to take. For example, when the children ate green vegetables four times, this was 4/5 (80%) of the recommended amount of green vegetables that week. Green vegetables are one of the four products and therefore composed 25% of the total dietary advice, so 80% of 25% = 20% of the total advice. The same calculation was used for the other food groups. The intake was calculated per week for 6 months. Parents of children in the control group recorded their children’s dietary routine via a grouped checklist for the products advised for the dietary advice group, which was less specific (e.g., what type of milk, butter, meat, or vegetables did they eat on a daily basis). The same method was used to calculate their weekly intake with respect to the parts of the dietary intake they spontaneously took. Diaries were collected and children were checked and measured at the clinic visits. Blood samples were obtained prior to the intervention and after six months at least seven days after an infectious episode. The complete blood count with differential, C-reactive protein, ferritin, immunoglobin A (IgA), IgG, IgM, IgE, zinc, and cholesterol profile were measured.

### 2.5. Outcome Measures

The primary outcome measures were (1) the number of days per month with two or more symptoms of an upper respiratory tract infection in the last three months of the study, assuming a certain delay for the diet to impact on immunity, gathered through the monthly diaries; (2) the total number of upper respiratory tract infections during the 6-month study period.

Secondary outcome measures were the number of days of parental absence from work due to a child with a URTI during the study, the number of prescribed antibiotics during the study, and differences in the total cholesterol: HDL-ratio between groups and over time (since full fat products are a major part of the dietary advice).

### 2.6. Sample Size

According to a previous pilot study, the hypothesis was that dietary advice could reduce the number of days with at least two symptoms of a URTI from 12 (±8) to 8 (±6) days per month [22]. This reduction was deemed achievable and clinically relevant in our patient population with recurrent URTIs. Calculations showed that 53 subjects were required in each group, with a power of 80% with two-sided testing at a significance level of 0.05, and assuming a non-normal distribution. A total of 120 patients were required for inclusion, assuming a 10% loss to follow up.

### 2.7. Statistical Methods

Descriptive statistics describe baseline demographic features. The primary and secondary outcome measures were analyzed according to their characteristics with a Mann–Whitney U, Wilcoxon signed rank, and independent or paired sample *t*-tests, as appropriate. Data are reported as mean (±SD) or a median (inter-quartile range, IQR) depending on the distribution. Some data (mean days on antibiotics and parental absence from work) were not normally distributed with median values are 0. In these cases, mean values with SD instead of median values are presented for interpretability, but tests were performed with Mann–Whitney U tests. For growth parameters, a mixed model analysis was used, where group and measurement were both added as fixed variables. The model we used for body mass index (BMI) SD = group measurement group × measurement. The interaction term group × measurement was added to analyze differences between groups over time. This was conducted for height, weight, weight for height, and BMI.

If, despite randomization, relevant differences in baseline characteristics between the two groups existed, these were analyzed for influence on the outcome measures. Baseline characteristics that differed between the study groups (*p* ≤ 0.10) were tested for an association with the primary outcome variable (the number of days per month with 2 or more symptoms of an URTI in the last 3 months of the study, which was highly skewed) and considered as potential confounders if *p* ≤ 0.10. To assess the relationship between continuous independent variables (e.g., age, BMI) and the outcome variable, a Spearman rank correlation analysis was performed. For categorical variables (e.g., sex) a Mann–Whitney U test was performed. Ultimately, as can be expected in a randomized controlled trial, no corrections for confounders were necessary. The analyses were conducted according to the modified intention-to-treat principle. Patients lost to follow up before the start of the first month of measuring were excluded from the analyses. A *p*-value of <0.05 was considered statistically significant. Statistical analyses were performed using the software IBM SPSS Statistics version 25 (IBM, New York, NY, USA).

## 3. Results

### 3.1. Population

Between March 2015 and October 2017, 156 patients with recurrent URTIs were eligible for inclusion after being examined by a pediatrician. A total of 125 patients (80%) agreed to participate and informed consent was provided by parent(s) or caregiver(s). These patients were randomized to the dietary advice plus supportive care (dietary advice group), or supportive care alone (control group). Their characteristics are listed in Table 1. No differences were found in the parental demographics in terms of educational attainment or socio-economic status. 

After inclusion, seven patients (6%, two in the intervention and five in the control group) dropped out during the first three months of the study and were not included in the analyses. The reasons for these exclusions were failure to record infectious episodes and symptoms in the diary (*n* = 3) or complete loss to follow up (*n* = 4). Their characteristics were similar compared with the subjects who completed the trial.

### 3.2. Adherence to Dietary Advice

The adherence to the dietary advice was 88% for the whole diet during the six months of the study. Adherence was lowest for green vegetables (82%) compared to beef, whole milk, and whole butter (92%, 90%, and 91%, respectively). The regular dietary intake (without dietary advice) of the control group reported by their diaries followed 34% of the dietary advice, mainly by their intake of beef and green vegetables. The regular dietary intake of the control group met 48% of the green vegetable advice, 60% of the beef advice, 10% of the whole milk, and 18% of the butter advice.

### 3.3. Outcome Measures

#### 3.3.1. Primary Outcome Measures: Duration and Number of Infections

The median (IQR) number of days per month with two or more symptoms of a URTI in the last three months of the study was 4.8 (1.6–9.5) in the dietary advice group compared to 7.7 (4.0–12.3) in the control group (*p* = 0.028). The total number of URTI episodes during the study period of 6 months was 5.7 (±0.55) in the intervention group compared to 6.8 (±0.49) episodes in the control group. The primary and secondary outcome measures are presented in Table 2.

Some data were not normally distributed (e.g., days on antibiotics and parental absence), and median values were zero. In these cases, mean values are presented in Table 2 for interpretability, but Mann–Whitney U tests were performed.

#### 3.3.2. Other Clinical Outcomes

The number of days with antibiotic use and the percentage of children who received antibiotic treatment during the six months of study were significantly lower in the dietary advice group. We noted a significant difference between groups in the number of days with fever in the last three months of the study; the median was 0.83 (0.0–1.7) in the dietary advice group versus 1.7 (0.7–3.0) in the control group (*p* < 0.01). The days the children coughed and suffered from a blocked nose were fewer in the intervention group (Table 2).

In the control group, parents visited their general practitioner more often during the six months with a mean of 0.9 (SD 1.5) visits in six months compared to 1.6 (SD 2.3) visits in the dietary advice group (Mann–Whitney U test, *p* = 0.031). Parents in the dietary advice group were not significantly less absent from work due to child illness.

#### 3.3.3. Other Outcome Measures 

To investigate whether the dietary advice would produce its effect by immunomodulation, we performed laboratory investigations. The only laboratory parameter that showed a between-group difference was CRP. In the dietary advice group, CRP decreased from 5.35 to 2.46 mg/L (*p* = 0.044), but remained unchanged in the control group (*p* = 0.82) with a difference between groups over time (*p* = 0.034), possibly suggestive of a lower grade of inflammation in the intervention group. The clinical meaning of this decrease should be used with caution since CRP values < 10 mg/L are regarded as non-elevated values. Other laboratory values did not differ between the groups after six months of follow up (Table S3).

Laboratory analyses showed that the cholesterol/HDL ratio was slightly lowered in both groups during the study (dietary advice group −0.56, *p* < 0.001); control group −0.36, *p* = 0.06) but not different between both groups (*p* = 0.40). Within groups, we observed some slight increases or decreases during the six months of follow up. In the dietary advice group, Hb, MCV, zinc, IgE, total cholesterol, and HDL increased, while leucocytes, lymphocytes, ferritin, IgM, and triglycerides decreased significantly compared with the starting values after six months of following the dietary advice. In the control group, zinc increased, and triglycerides and lymphocytes decreased significantly for the same period (Table S3).

We found no significant changes in height or weight gain between groups. Table 3 provides full details on biometry values. No harmful or unintended effects were noted during the study.

## 4. Discussion

The dietary advice significantly decreased the number of days with a URTI and taking antibiotics as well as the number of children using antibiotics, and symptoms of coughing, fever, and blocked nose. No difference in the number of URTIs was observed. This suggests a shorter duration of infection. The diet did not reduce the number of days of parental absence from work. The study was, however, not designed to detect differences between groups in these secondary outcome measurements. Laboratory parameters did not significantly change over time between the two groups to explain the observed clinical differences.

The main strength of this study is the uniqueness of applying a randomized controlled trial (RCT) design to evaluate the effects of a full diet intervention. We tried to adhere to the most frequently used outcome measures in nutritional interventions in children with respiratory infections, as indicated by the COMMENT Initiative as well as daily questionnaires on common cold symptoms [25,27]. We incorporated a RCT design with normal eating practices, providing valid reflection of possible effects on daily life [28]. Our dietary advice was based on the qualities of the food matrix and food synergy in relation to fresh, locally produced, and traditional food. The food matrix is based on structural capacities of foods, in which macro and micronutrients are offered. This food structure makes the difference between biological and health outcomes [29]. Food synergy is based on the proposition that the interrelations between constituents in foods are important, as well as the interactions between foods, and that the total effect on health is greater than just the sum of all individual effects [14]. The studies mentioned in the introduction focused on a limited number of supplementations, each with their own working mechanism [10,11,12,13]. Our dietary advice provides the proper nutritional values while leaving the food matrix intact. However, the specific mechanisms of action are still unknown.

The dietary advice was based on food composition derived from the Dutch Food Composition Database [20]. The diet contains proper quantities of vitamins A, C, D, and E, zinc, iron, and fatty acids (Table S1). All these components show potential to positively influence the immune response in children. Numerous immunological effects of various nutrients have been published as well as the routing of the activated immune cells to the airways, thereby linking the dietary components to chronic inflammation by means of the intestine–airway axis [12,30,31,32,33].

Studies with RCTs and whole foods for URTI are scarce. Positive effects on the duration or prevention of URTIs in adults or children were observed with the intake of probiotics, prebiotics, milk, fish oil, kiwi fruit, garlic, xylitol, and elderberry [34,35,36,37,38,39,40,41]. The studies focused on a single whole food product, not a combination of food products. In another study, the combination of food products was investigated with the Mediterranean diet. In a before–after comparison after the adoption of the Mediterranean diet, Calatayud et al. found a reduction in URTIs from 7.45 ± 1.74 to 2.88 ± 1.60 episodes over one year in children aged one to five years [15]. Due to the absence of a control group, it was hard to assess causality, since the number of infections declines with advancing age [1].

Due to public opinion, parents are sometimes afraid about possible side effects of adhering to a dietary advice including beef and whole dairy products. Red meat has been under scrutiny due to possible carcinogenic attributes. The role of beef in this matter remains questionable, since red meat is a catch-all for beef, pork, veal, lamb, horse, etc. The carcinogenic role of meat seems to be more applicable to processed meat instead of red meat [42]. However, a metanalysis found low certainty evidence of harmful effects of red meat in general and concluded that adults can continue to consume both unprocessed and processed red meat [43].

Our results showed that consuming whole dairy products does not increase the cholesterol/HDL ratio during six months of following this dietary advice, is in line with other reports [44,45]. Consumption of dairy products has been associated with a lower risk of mortality and major cardiovascular disease events in a diverse multinational cohort, without added risk by intake of whole fat dairy products [46]. These findings are consistent with insights that fats from animal origin (intake from fats by dairy or meat consumption) increase HDL levels and decrease the cholesterol/HDL ratio [47]. We found no increased weight gain or increase in BMI after consuming whole dairy products, beef and green vegetables. This corresponds to a large meta-analysis that also found no increase in obesity in children drinking whole milk [48]. The strength of our study is that we found no increase in obesity after consuming whole milk in a clinical trial, which confirms the observational studies. However, our study lasted 6 months, a study with a longer follow-up could provide more clarity about long-term growth.

Some limitations must be considered. The loss to follow up was slightly greater in the control group, but the number of patients dropping out was small (3% vs. 8%). We therefore think that these patients did not influence the results.

Blinding of participants to the dietary advice was impossible. Although parents in the control group were not aware about the specifics of the dietary advice, it is possible that subjects in the control group may had heard about it due to earlier studies by the primary investigator. They may have chosen to incorporate (parts of) the diet into their daily lives. Data from the diaries of both groups showed this influence to be limited: Children in the intervention group adhered to the dietary advice by 89% versus 34% in the control group. We interpreted the diet of the control group as normal child eating habits in our region. Possible contamination bias in the control group would therefore only complicate the detection of differences between groups.

The finding that fewer antibiotic courses were prescribed and a lower number of visits to the general practitioner was reported in the dietary advice group may also be influenced by not blinding participants. Parents may have been influenced by knowing they were in the group receiving treatment and therefore were less demanding of antibiotic treatment when visiting a physician.

### Implications for Research and/or Practice

Our results showed a significant reduction of days with URTIs and symptoms but failed to reduce the number of episodes with a URTI. This could be due to an assumed delay for the diet to impact immunity, and more time is needed to observe the impact on the number of infections. On the other hand, it can also suggest that adjusting the dietary intake can influence the course or duration of infections rather than preventing the child from falling ill. The Cochrane review on vitamin C also showed beneficial effects of vitamin C on the common cold [12]. We do not think that the effect of the investigated dietary advice is solely explained by the vitamin C content of the diet, since other food products low in vitamin C also shorten the duration of URTI in children [34,35,37,40]. We think that the combination of products has its operating mechanism in its full spectrum. Overall, exposure to certain viral pathogens in childhood is necessary for achieving a proper immune function. Normal exposure to pathogens and decreasing severity of contracted illness shows the adaptability that reflects a good state of health [49].

Since healthcare costs are high for URTI and several often ineffective antibiotic treatments are prescribed, dietary intervention can potentially reduce healthcare costs [8] through reducing the use of antibiotics and visits to the general practitioner. Due to decreased antibiotic prescriptions, the treatment could help reduce antibiotic resistance or response failure in the future.

Our findings can further strengthen the scientific evidence supporting national and global nutritional guidelines. The low-cost nature of the intervention makes the dietary advice easily applicable for widespread preventive care for parents. The costs are a little higher due to the higher costs of beef compared to chicken or pork. Due to the small portion sizes consumed by young children, the estimated extra costs in the Netherlands are €0.67 per week. Since no treatment exists for children suffering from URTI without immunological disorders, this diet provides parents with a tool to improve the health of their children.

## 5. Conclusions

We found that a diet of green vegetables, beef, and whole dairy products in children with recurrent URTIs can decrease the number of days per months with multiple symptoms of a URTI. The dietary advice also leads to fewer prescribed antibiotics and can therefore be a possible tool to help reduce antibiotic prescriptions.

## Figures and Tables

**Table 1:** Baseline characteristics of the subjects in the dietary advice and control group with median with interquartile range or mean ± SD.

Variable	Dietary Advice (*n* = 58)	Control Group (*n* = 60)
**Personal characteristics**		
Age (years)	2.4 ± 1.1	2.4 ± 1.0
Girls (%)	21 (37%)	36 (63%)
BMI-for-age standard score	−0.13 ± 1.16	−0.06 ± 1.26
Height-for-age standard score	0.02 ± 1.20	−0.25 ± 0.89
**Perinatal characteristics**		
Delivery (vaginal)	33 (46%)	39 (54%)
Gestational age (weeks)	39.0 (38–40)	38.5 (38–40)
Birth weight (kg)	3.5 ± 0.6	3.2 ± 0.7
Duration of breastfeeding (months)	1 (0–3)	1 (0–3)
**Clinical characteristics**		
Two or more symptoms of URTI in the run-in period (days/month)	14.5 ± 7.9	14.4 ± 9.0
Infection episodes in run in period (*n*/month)	2.0 (1.0–3.0)	1.5 (1.0–2.0)
Day care attendance (days/week)	2.0 ± 1.5	1.9 ± 1.5
Parental smoking	35 (60%)	31 (52%)
Number of children (%) with 1 or more antibiotic treatments in run in period	14 (25%)	13 (22%)

BMI, body mass index; URTI, upper respiratory tract infection; mean values = age, BMI-for-age standard score, height-for-age standard score, birthweight, URTI symptoms in run-in period, day care attendance; median values = gestational age, infection episodes run-in period.

**Table 2:** Primary and secondary outcome measures and their relevant components in the dietary advice group and control group.

Characteristic	Dietary Advice (*n* = 58)	Control Group (*n* = 60)	RR (95% CI), Mean Difference (95% CI, or *p*-Value
**Primary outcome measurements**			
Two or more symptoms of an URTI (days/month) In the last 3 months of the study (median, IQR)	4.8 (1.5–9.5)	7.7 (4.0–12.3)	*p* = 0.028 ^a^
No. of infection periods/month (mean, SD)	0.95 (±0.55)	1.13 (±0.49)	−0.2 (−0.39; 0.1) ^c^
**Secondary outcome measurements**			
Mean days on antibiotics in 6 months (mean, SD)	2.9 (±12.7)	5.0 (±9.4)	*p* = 0.002 ^b^
Number of children (%) with 1 or more antibiotic treatments in 6 months	10 (17.2%)	27 (45%)	0.39 (0.20; 0.72) ^d^
Days of parental absence of work in 6 months (mean, SD)	0.2(±0.45)	0.3(±0.66)	*p* = 0.893 ^b^
**Symptoms in last 3 months;** **no. of days/month**			
Coughing (mean, SD)	5.3 (±4.9)	8.2 (±6.0)	−3.0 (−5.0; −1.0) ^c^
Fever (mean, SD)	1.2 (±1.4)	2.1 (±1.9)	−0.9 (−1.5; −0.3) ^c^
Blocked nose (mean, SD)	2.5 (±3.9)	4.6 (±5.8)	−2.1 (−3.9; −0.3) ^c^
Runny nose (mean, SD)	6.2 (±6.2)	8.0 (±7.0)	−1.8 (−4.2; 0.65) ^c^
Sore throat (mean, SD)	1.0 (±2.0)	1.1 (±1.7)	−0.1 (−0.8; 0.58) ^c^
Wheezing (mean, SD)	3.5 (±6.7)	4.0 (±7.2)	−0.6 (−3.1; 2.0) ^c^
Otitis (mean, SD)	1.6 (±5.1)	0.6 (±1.6)	0.9 (−0.45; 2.3) ^c^

URTI, upper respiratory tract infection; HDL, high density lipoprotein; ^a^ continuous variable, not normally distributed, with median > 0; median (IQR) and *p*-value of Mann–Whitney U test are presented. ^b^ continuous variables, not normally distributed, with median = 0; mean (SD) and *p*-value of Mann–Whitney U test are presented. ^c^ continuous variables, normally distributed; mean (SD) and mean difference (95% CI) are presented. ^d^ categorical variables, relative risk (RR) and 95% CI are presented.

**Table 3:** Biometrics of participants (SD according to standardized Dutch growth graphs).

Parameter	Dietary Advice			Control Group			*p*-Value
	*T* = 0	*T* = 3	*T* = 6	*T* = 0	*T* = 3	*T* = 6	
Weight in SD	−0.101	−0.061	−0.053	−0.231	−0.141	−0.089	0.6
Height in SD	0.023	0.086	0.052	−0.228	−0.217	−0.295	0.6
Weight for height in SD	−0.074	−0.072	−0.020	−0.073	0.016	0.178	0.4
BMI in SD	−0.136	−0.111	−0.098	−0.061	0.038	0.207	0.14

Mixed model analysis. *p*-values indicate the group × measurement interaction.

## Supplementary Materials

The following are available online at https://www.mdpi.com/2072-6643/12/1/272/s1. Table S1. Average daily nutritional values from the dietary advice alone ^19^. This does not contain additional nutrients from other meals, snacks and beverages, Table S2. The dietary advice with age specific portion sizes ^20^, Table S3. Laboratory values of both groups at baseline (T0). at 6 months (T6) and *p*-values for differences between groups and in time.

## References

[1] Gruber C., Keil T., Kulig M., Roll S., Wahn U., Wahn V., M.A.S.S. Group (2008). History of respiratory infections in the first 12 yr among children from a birth cohort. Pediatr. Allergy Immunol..

[2] Heikkinen T., Jarvinen A. (2003). The common cold. Lancet.

[3] Turner R.B. (1997). Epidemiology, pathogenesis, and treatment of the common cold. Ann. Allergy Asthma Immunol..

[4] Ball T.M., Holberg C.J., Aldous M.B., Martinez F.D., Wright A.L. (2002). Influence of attendance at day care on the common cold from birth through 13 years of age. Arch. Pediatr. Adolesc. Med..

[5] De Vries E., Kuijpers T.W., van Tol M.J., van der Meer J.W., Weemaes C.M., van Dongen J.J. (2000). Immunology in medical practice. XXXV. Screening of suspected immunodeficiency: Diagnostic protocols for patients with opportunistic or recurrent severe infections, wasting and failure to thrive. Ned. Tijdschr. Geneeskd..

[6] Lee G.M., Friedman J.F., Ross-Degnan D., Hibberd P.L., Goldmann D.A. (2003). Misconceptions about colds and predictors of health service utilization. Pediatrics.

[7] Bramley T.J., Lerner D., Sames M. (2002). Productivity losses related to the common cold. J. Occup. Environ. Med..

[8] Mainous A.G., Hueston W.J. (1998). The cost of antibiotics in treating upper respiratory tract infections in a medicaid population. Arch. Fam. Med..

[9] Van Hecke O., Fuller A., Bankhead C., Jenkins-Jones S., Francis N., Moore M., Butler C., Wang K. (2019). Antibiotic exposure and ‘response failure’ for subsequent respiratory tract infections: An observational cohort study of UK preschool children in primary care. Br. J. Gen. Pract..

[10] Yakoob M.Y., Salam R.A., Khan F.R., Bhutta Z.A. (2016). Vitamin D supplementation for preventing infections in children under five years of age. Cochrane Database Syst. Rev..

[11] Jonker F.A., van Hensbroek M.B. (2014). Anaemia, iron deficiency and susceptibility to infections. J. Infect..

[12] Hemila H., Chalker E. (2013). Vitamin C for preventing and treating the common cold. Cochrane Database Syst. Rev..

[13] Martineau A.R., Jolliffe D.A., Hooper R.L., Greenberg L., Aloia J.F., Bergman P., Dubnov-Raz G., Esposito S., Ganmaa D., Ginde A.A. (2017). Vitamin D supplementation to prevent acute respiratory tract infections: Systematic review and meta-analysis of individual participant data. BMJ.

[14] Jacobs D.R., Gross M.D., Tapsell L.C. (2009). Food synergy: An operational concept for understanding nutrition. Am. J. Clin. Nutr..

[15] Calatayud F.M., Calatayud B., Gallego J.G., Gonzalez-Martin C., Alguacil L.F. (2017). Effects of Mediterranean diet in patients with recurring colds and frequent complications. Allergol. Immunopathol..

[16] Fondell E., Christensen S.E., Balter O., Balter K. (2011). Adherence to the Nordic Nutrition Recommendations as a measure of a healthy diet and upper respiratory tract infection. Public Health Nutr..

[17] Huijbregts P.P., Feskens E.J., Rasanen L., Alberti-Fidanza A., Mutanen M., Fidanza F., Kromhout D. (1995). Dietary intake in five ageing cohorts of men in Finland, Italy and The Netherlands. Eur. J. Clin. Nutr..

[18] FAO (2019). Food Balance Sheets. http://www.fao.org/faostat/en/#home.

[19] Munow M., van der Gaag E.J. Ailing Toddlers. Is there a relation between behaviour and health?. Proceedings of the 27th ESPID Meeting.

[20] NEVO-tables Dutch Food Composition Database. https://nevo-online.rivm.nl/.

[21] Voedingscentrum (2019). Example Diet for Young Children, The Netherlands Nutrition Centre. https://www.voedingscentrum.nl/nl/mijn-kind-en-ik/dreumes-en-peuter/voorbeelddagmenu-voor-dreumes-en-peuter.aspx.

[22] Ten Velde L.G.H., Leegsma J., van der Gaag E.J. (2013). Recurrent Upper Respiratory Tract Infections in Children: The Influence of Green Vegetables, Beef, Whole Milk and Butter. Food Nutr. Sci..

[23] Saghaei M., Saghaei S. (2011). Implementation of an open-source customizable minimization program for allocation of patients to parallel groups in clinical trials. J. Biomed. Sci. Eng..

[24] Chatchatee P., Lee W.S., Carrilho E., Kosuwon P., Simakachorn N., Yavuz Y., Schouten B., Graaff P.L., Szajewska H. (2014). Effects of growing-up milk supplemented with prebiotics and LCPUFAs on infections in young children. J. Pediatr. Gastroenterol. Nutr..

[25] Eccles R. (2013). Is the common cold a clinical entity or a cultural concept?. Rhinology.

[26] Burke L., Stifano T. (2006). Guidance for industry: Patient-reported outcome measures: Use in medical product development to support labeling claims: Draft guidance. Health Qual. Life Outcomes.

[27] Guarino A., Bruzzese E., Vecchio A.L., Dagan R., Tsolia M. (2013). Definitions and outcomes of nutritional interventions in children with respiratory infections: The approach of the COMMENT initiative. Ann. Nutr. Metab..

[28] Penders B., Wolters A., Feskens E.F., Brouns F., Huber M., Maeckelberghe E.L.M., Navis G., Ockhuizen T., Plat J., Sikkema J. (2017). Capable and credible? Challenging nutrition science. Eur. J. Nutr..

[29] Wahlqvist M.L. (2016). Food structure is critical for optimal health. Food Funct..

[30] Hassan T.H., Badr M.A., Karam N.A., Zkaria M., el Saadany H.F., Rahman D.M.A., Shahbah D.A., al Morshedy S.M., Fathy M., Esh A.M. (2016). Impact of iron deficiency anemia on the function of the immune system in children. Medicine.

[31] Jimenez C., Leets I., Puche R., Anzola E., Montilla R., Parra C., Aguilera A., Garcia-Casal M.N. (2010). A single dose of vitamin A improves haemoglobin concentration, retinol status and phagocytic function of neutrophils in preschool children. Br. J. Nutr..

[32] Perdijk O., van Splunter M., Savelkoul H.F.J., Brugman S., van Neerven R.J.J. (2018). Cow’s Milk and Immune Function in the Respiratory Tract: Potential Mechanisms. Front. Immunol..

[33] Van Splunter M., van Hoffen E., Floris-Vollenbroek E.G., Timmerman H., de Bos E.L., Meijer B., Ulfman L.H., Witteman B., Wells J.M., Brugman S. (2018). Oral cholera vaccination promotes homing of IgA(+) memory B cells to the large intestine and the respiratory tract. Mucosal Immunol..

[34] Hao Q., Dong B.R., Wu T. (2015). Probiotics for preventing acute upper respiratory tract infections. Cochrane Database Syst. Rev..

[35] Hughes C., Davoodi-Semiromi Y., Colee J.C., Culpepper T., Dahl W.J., Mai V., Christman M.C., Langkamp-Henken B. (2011). Galactooligosaccharide supplementation reduces stress-induced gastrointestinal dysfunction and days of cold or flu: A randomized, double-blind, controlled trial in healthy university students. Am. J. Clin. Nutr..

[36] Li F., Jin X., Liu B., Zhuang W., Scalabrin D. (2014). Follow-up formula consumption in 3- to 4-year-olds and respiratory infections: An RCT. Pediatrics.

[37] Thienprasert A., Samuhaseneetoo S., Popplestone K., West A.L., Miles E.A., Calder P.C. (2009). Fish oil n-3 polyunsaturated fatty acids selectively affect plasma cytokines and decrease illness in Thai schoolchildren: A randomized, double-blind, placebo-controlled intervention trial. J. Pediatr..

[38] Adaim A. (2010). Investigating the Effect of Gold Kiwifruit Consumption on the Incidence and Symptoms of Upper Respiratory Tract Infections in Pre-School Children. Ph.D. Thesis.

[39] Lissiman E., Bhasale A.L., Cohen M. (2014). Garlic for the common cold. Cochrane Database Syst. Rev..

[40] Azarpazhooh A., Lawrence H.P., Shah P.S. (2016). Xylitol for preventing acute otitis media in children up to 12 years of age. Cochrane Database Syst. Rev..

[41] Zakay-Rones Z., Thom E., Wollan T., Wadstein J. (2004). Randomized study of the efficacy and safety of oral elderberry extract in the treatment of influenza A and B virus infections. J. Int. Med. Res..

[42] Johnson I.T. (2017). The cancer risk related to meat and meat products. Br. Med. Bull..

[43] Johnston B.C., Zeraatkar D., Han M.A., Vernooij R.W.M., Valli C., el Dib R., Marshall C., Stover P.J., Fairweather-Taitt S., Wojcik G. (2019). Unprocessed Red Meat and Processed Meat Consumption: Dietary Guideline Recommendations From the Nutritional Recommendations (NutriRECS) Consortium. Ann. Intern. Med..

[44] Engel S., Elhauge M., Tholstrup T. (2018). Effect of whole milk compared with skimmed milk on fasting blood lipids in healthy adults: A 3-week randomized crossover study. Eur. J. Clin. Nutr..

[45] Van der Gaag E.J., Wieffer R., van der Kraats J. (2017). Advising Consumption of Green Vegetables, Beef, and Full-Fat Dairy Products Has No Adverse Effects on the Lipid Profiles in Children. Nutrients.

[46] Dehghan M., Mente A., Rangarajan S., Sheridan P., Mohan V., Iqbal R., Gupta R., Lear S., Wentzel-Viljoen E., Avezum A. (2018). Prospective Urban Rural Epidemiology study, Association of dairy intake with cardiovascular disease and mortality in 21 countries from five continents (PURE): A prospective cohort study. Lancet.

[47] Liu Y., Poon S., Seeman E., Hare D.L., Bui M., Iuliano S. (2019). Fat from dairy foods and ‘meat’ consumed within recommended levels is associated with favourable serum cholesterol levels in institutionalised older adults. J. Nutr. Sci..

[48] Vanderhout S.M., Aglipay M., Torabi N., Juni P., da Costa B.R., Birken C.S., O’Connor D.L., Thorpe K.E., Maguire J.L. (2019). Whole milk compared with reduced-fat milk and childhood overweight: A systematic review and meta-analysis. Am. J. Clin. Nutr..

[49] Huber M., Bakker M.H., Dijk W., Prins H.A., Wiegant F.A. (2012). The challenge of evaluating health effects of organic food; operationalisation of a dynamic concept of health. J. Sci. Food Agric..

