# Supplementary material for: Influence of Dietary Advice Including Green Vegetables, Beef, and Whole Dairy Products on Recurrent Upper Respiratory Tract Infections in Children: A Randomized Controlled Trial
Source: Nutrients. 2020 Jan 20;12(1):272. doi: 10.3390/nu12010272 (PMC7019298; doi:10.3390/nu12010272)
Supplement: Supplementary file 1 [file nutrients-12-00272-s001.pdf]

SUPPLEMENTAL MATERIALS

<table>
<tr><td></td><td colspan="2">Vit A (μg)</td><td colspan="2">Vit C (mg)</td><td colspan="2">Vit D (μg)</td><td colspan="2">Vit E (mg)</td><td colspan="2">Zinc (mg)</td><td colspan="2">Iron (mg)</td></tr>
<tr><td></td><td>Diet</td><td>RDA</td><td>Diet</td><td>RDA</td><td>Diet</td><td>RDA</td><td>Diet</td><td>RDA</td><td>Diet</td><td>RDA</td><td>Diet</td><td>RDA</td></tr>
<tr><td>Minimum</td><td>275</td><td rowspan="2">400</td><td>14</td><td rowspan="2">40</td><td>0.9</td><td rowspan="2">10*</td><td>1.9</td><td rowspan="2">5.5</td><td>4.7</td><td rowspan="2">4</td><td>2.1</td><td rowspan="2">7</td></tr>
<tr><td>Maximum</td><td>445</td><td>18</td><td>0.9</td><td>2.6</td><td>7.9</td><td>2.8</td></tr>
</table>

* Supplementation according to national guidelines. RDA = recommended daily allowance.

Table S1. Average daily nutritional values from the dietary advice alone[19]. This does not contain additional nutrients from other meals, snacks and beverages.

**Product**	**Frequency**	**Portion size (1-4y)**
Green vegetables	5 times per week	50-100 g
Beef	3 times per week	50-60 g
Bovine milk	Daily	300 mL
Whole dairy butter on bread	Daily	5 g/slice

Table S2. The dietary advice with age specific portion sizes[20].

Table S3. Laboratory values of both groups at baseline (T0). at 6 months (T6) and p-values for differences between groups and in time.

	**Dietary Advice N=58**			**Control Group N=60**			**p-value** (between groups)
	T=0	T=6	p-value	T=0	T=6	p-value	
Hb (mmol/l)	7.34	7.59	<0.001	7.36	7.54	0.003	0.4
MCV (fl)	77.9	78.8	0.007	78.5	78.9	0.18	0.3
Leucocytes ($x10^9$)	10.3	9.26	0.024	9.35	8.88	0.36	0.3
Lymphocytes ($x10^9$)	4.91	4.34	0.007	4.81	4.15	0.019	0.8
Neutrophils ($x10^9$)	4.04	3.88	0.41	3.34	3.57	0.54	0.3
CRP (mg/l)	5.35	2.46	0.044	2.98	3.02	0.83	0.034
Ferritin (ug/l)	43.3	33.0	0.08	51.6	41.5	0.38	0.7
Zinc (umol/l)	11.2	12.8	<0.001	11.5	12.3	0.033	0.2
IgA (g/l)	0.62	0.58	0.82	0.67	0.68	0.27	0.5
IgG (g/l)	7.25	7.21	0.73	7.74	7.49	0.40	0.7
IgM (g/l)	0.97	0.89	0.018	0.96	0.89	0.48	0.4
IgE (g/l)	33.9	58.9	0.004	37.1	62.0	0.31	0.16
Cholesterol (mmol/l)	3.99	4.12	0.047	3.97	3.97	0.93	0.20
Triglycerides (mmol/l)	1.46	1.08	0.005	1.45	1.20	0.042	0.6
HDL (mmol/l)	1.11	1.29	<0.001	1.14	1.21	0.16	0.11
LDL (mmol/l)	2.22	2.35	0.05	2.29	2.28	0.85	0.20
Cholesterol/HDL ratio	3.95	3.32	<0.001	3.77	3.47	0.06	0.4

Normally distributed, student -test and mean values; Hb, MCV, leucocytes, lymphocytes, Neutrophils, IgA, IgG, Cholesterol, triglycerides, HDL, LDL, Cholesterol/HDL ratio

Not normally distributed, Mann Whitney test and median values; CRP, ferritin, IgM, IgE
